# A Study of the S-1 or Capecitabine as First-line Regimen in Patients with Metastatic Colorectal Cancer: A Real World Study

**DOI:** 10.7150/jca.36929

**Published:** 2020-01-20

**Authors:** Yanan Guo, Tongsen Zheng, Chunhui Zhang, Yanqiao Zhang

**Affiliations:** 1 Department of Gastrointestinal Medical Oncology, Harbin Medical University Cancer Hospital, Institute of Prevention and Treatment of Cancer of Heilongjiang Province, Harbin Heilongjiang 150081, China.; 2 Department of Phase I Clinical Trials, Harbin Medical University Cancer Hospital.

**Keywords:** S-1, capecitabine, real-world, metastatic colorectal cancer, first-line treatment

## Abstract

**Objectives:** To compare the 2-year overall survival (OS) rate and safety between patients using S-1 and capecitabine in the first-treatment of metastatic colorectal cancer in the real clinical setting.

**Methods:** In this retrospective cohort study, patients satisfying the following criteria were identified from 10 centers in China. The 2-year OS rate and safety were assessed. The propensity score matching (PSM) was used to control basic characteristics of the two groups to balance the processing bias and confoundings.

**Results:** A total of 1367 patients were identified, 824 patients accepted capecitabine and 546 patients accepted S-1. After PSM, 533 eligible patients were included in each group without statistical significance in age, sex, BMI, KPS score and comorbidities. The 2-year OS rate between two groups was without significant statistical difference (61.9% vs. 62.9%, p=0.4295). The subgroup analysis showed that the 2-year OS rate had no significant difference between men and women, younger and older than 60 years old, different metastatic sites, different chemotherapy courses between S-1 and capecitabine groups. The hematological adverse events were all without statistical difference between two groups, but the incidence of diarrhea (16.4% vs. 23.6%, p=0.0018) and hand-foot syndrome (28.7% vs. 46.7%, p<0.001) in S-1 group were lower than those in the capecitabine group.

**Conclusions:** Compared to capecitabine, S-1 had a similar 2-year OS rate but had a lower incidence of adverse events in the real clinical setting. So, S-1 could be a good choice in the first-treatment of patients with metastatic colorectal cancer in China.

## Introduction

Colorectal cancer, as one of the most common types of malignant tumors, is often asymptomatic in early stage and up to 25% of patients have already developed metastatic colorectal cancer (mCRC) at the first diagnosis 1. Double combination chemotherapy plus targeted agents remain as the mainstay treatment for mCRC, and oxaliplatin plus infusional fluorouracil and leucovorin (FOLFOX) has been considered the standard regimen 2-3. In response to the need for new therapeutic options offering improved efficacy, tolerability and convenience for patients instead of intravenous 5-fluorouracil, the new oral fluoropyrimidines have been developed. The oral fluoropyrimidine capecitabine has been demonstrated that as first-line therapy for mCRC to be non-inferior to 5-Fu, which achieves significantly superior response rates, equivalent time to disease progression and equivalent survival 4-5. However,reported rates of hand-foots syndrome (HFS) are higher with capecitabine 6. S-1, is an oral fluoropyrimidine that is composed of tegafur with two modulators of 5-Fu metabolism, 5-chloro-2,4-dihydrogenase (CDHP), and oteracil potassium. Oteracil potassium decreases incorporation of 5-fluorouridine triphosphate into RNA in the gastrointestinal mucosa and reduces incidence of diarrhea. CDHP is a reversible inhibitor of dihydropyrimidine dehydrogenase(DPD). It has been suggested that HFS is elicited by 5-Fu catabolites. When DPD-inhibitors are added to fluoropyrimidines or patients with a DPD-deficiency, the incidence of HFS are lower. Therefore, S-1 may decrease the incidence of neurotoxicity and cardiotoxicity 7-9. S-1 has comparable efficacy results compared to capecitabine in gastric cancer by several trials in Japan and Korea 10-12. Some phase II/III trials demonstrated that S-1 was as efficacious as capecitabine with respect to overall survival (OS) and progression-free survival (PFS) in mCRC and reduced the frequency of adverse events 13-17 We intent to compare the efficacy and safety of S-1 (Tegafur Gimeracil and Oteracil Potassium Capsules, ShanDong Newtime Pharmaceutical Co.Ltd) or Capecitabine Tablets (Xeloda, F.Hoffmann-La Roche, Basel, Switzerland) monotherapy or doublet chemotherapy (including oxaliplatin, irinotecan, raltitrexed, elemene and others) as first-line treatment in mCRC patients in real world study in China.

## Materials and Methods

### Study design

This was a multi-center, retrospective observational study conducted at 10 centers including the Harbin Medical University Cancer Hospital, the First Affiliated Hospital of Harbin Medical University, the LiaoNing Cancer Hospital & Institute, the General Hospital of Shenyang Military, AnYang Tumor Hospital, Haikou People's Hospital, Hospital of HeBei Medical University, HeNan Cancer Hospital, JiangSu Cancer Hospital, and Affiliated Hospital of JiangSu University. It aims to compare the efficacy and safety between two groups as the first-line treatment in patients with mCRC in a real clinical setting. Patients were eligible for this study if they had metastatic colorectal cancer (the primary cancer) and had not received prior chemotherapy for metastatic disease. After screening to establish eligibility, patients were naturally divided into the S-1 group and Capecitabine group according to real clinical treatment. The electronic medical record should be collected over a continuous time to avoid the bias caused by confounding factors as much as possible. The primary objective is comparing the 2-year overall survival rate of S-1 or Capecitabine monotherapy or doublet chemotherapy as first-line treatment in patients with mCRC in a real clinical setting. Secondary endpoints included the safety including myelosuppression (blood-related indicators), liver and kidney toxicity (related indicators of liver and kidney function), cardiotoxicity (electrocardiogram), gastrointestinal side effects (Nausea and Vomiting, Diarrhea) and hand-foot syndrome, namely grade 3/4.

### Patients

Patients were required to have the following criteria: 1) pathological diagnosis of metastatic colorectal cancer; 2) patients without prior chemotherapy or with S-1 or Capecitabine as first-line treatment for recurrence after adjuvant chemotherapy; 3) receive S-1or Capecitabine monotherapy or doublet chemotherapy at least 1 cycle of treatment; 4) not perform radiotherapy for target lesions during the treatment; 5) expected survival greater than 3 months. Exclusion criteria: 1) patients treated with immunotherapy or targeted therapy concurrent with chemotherapy; 2) patients with radical surgery or local treatment during chemotherapy; 3) patients accompanied by a second primary tumor. Based on the clinician's experiential suggestion, demographics, medical history, disease factors, chemotherapy cycle, as well as other critical factors influencing the prognosis of patients should be included as baseline characteristics. If there are systematic differences in the distribution of baseline characteristics between two groups, propensity score methods are an appropriate tool for the analysis of this study which can minimize the effect of confounding and reduce the bias.

### Statistical analysis

According to different analytical purposes, the set of patients whose data are to be included in the final analyses. Patients who are diagnosed with mCRC between June 2013 and June 2016 in 10 centers above, treated by S-1 or Capecitabine monotherapy or doublet chemotherapy as first-line chemotherapy, eligible for the inclusion and exclusion criteria with at least one safety observation and have follow-up data should be included. Consider that the prevalence of missing data in a retrospective study, analysis would be performed on the non-missing data. For matching variables, missing data should be excluded. All the variables will be summarized using descriptive statistics by treatment group. For categorical variables, frequency and percentage for each category will be presented. For continuous variables, the number of observation, mean, standard deviation (SD), median, minimum and maximum will be presented. 95% confidence interval will be provided if necessary. All statistical hypothesis tests were performed at two-sided significance level 0.05. We used SAS (version 9.4) for all statical analyses. The OS in two groups will be presented graphically by the Kaplan-Meier curve.

## Results

### Patients

**Figure 1:**
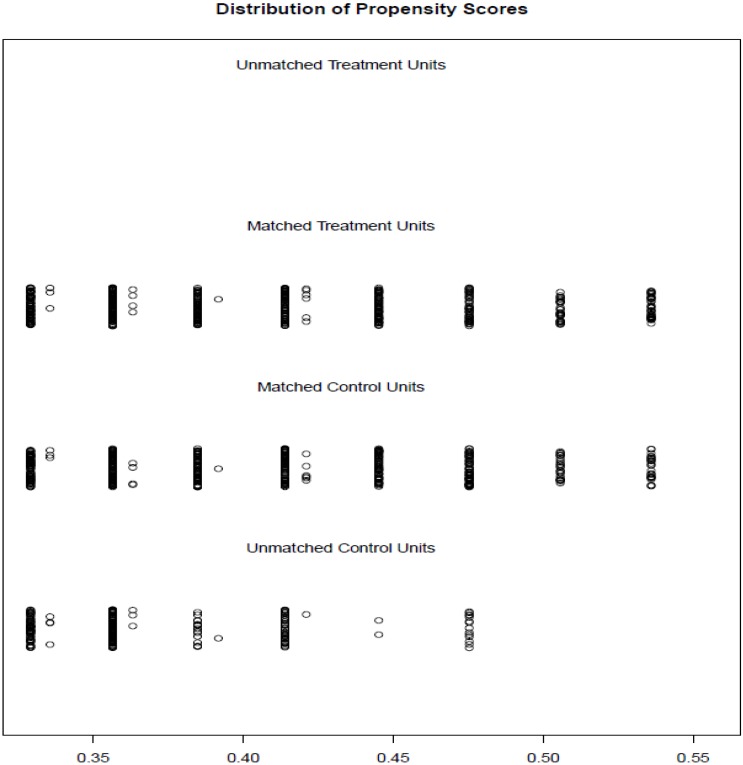
The distribution of propensity score

**Figure 2:**
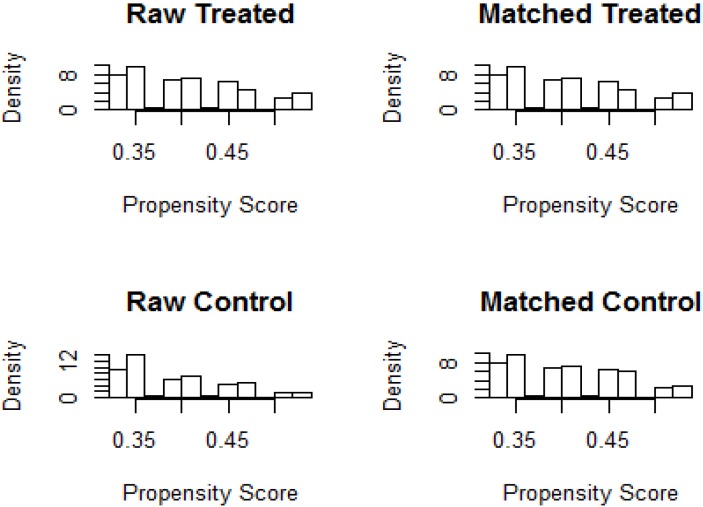
The histogram bar of propensity score

According to the inclusion and exclusion criteria, 1367 patients were eligible, including 824 males and 543 females with a median age of 60 years. Among them, 546 cases were in the S-1 group, 315 males and 231 females, with a median age of 60 years; 821 cases of the Capecitabine group, 509 males and 312 females, with a median age of 60 years (Table 1). Patients, if adverse reactions occur such as myelosuppression, gastrointestinal side effects and hand- foot syndrome, should be treated immediately. There was no statistically significance in gender, age, BMI and KPS scores between the two groups. The male to female ratio is about 3: 2; consider the median age (60 years), the number of patients with age older than 60 and less than 60 is similar. The BMI of patients with colorectal cancer was mostly between 18.5 and 28, and the KPS score was the highest at 80 and 90. Before matching, all the observations with missing values of matching variables would be eliminated. 1:1 Nearest Matching should be appropriate for the sample ratio of two groups is close to 1:1 and would be implemented by R3.3.1 software (Figure 1,2). The final matching result was 533 people in each group (Table 2,3).

### Efficacy

**Figure 3:**
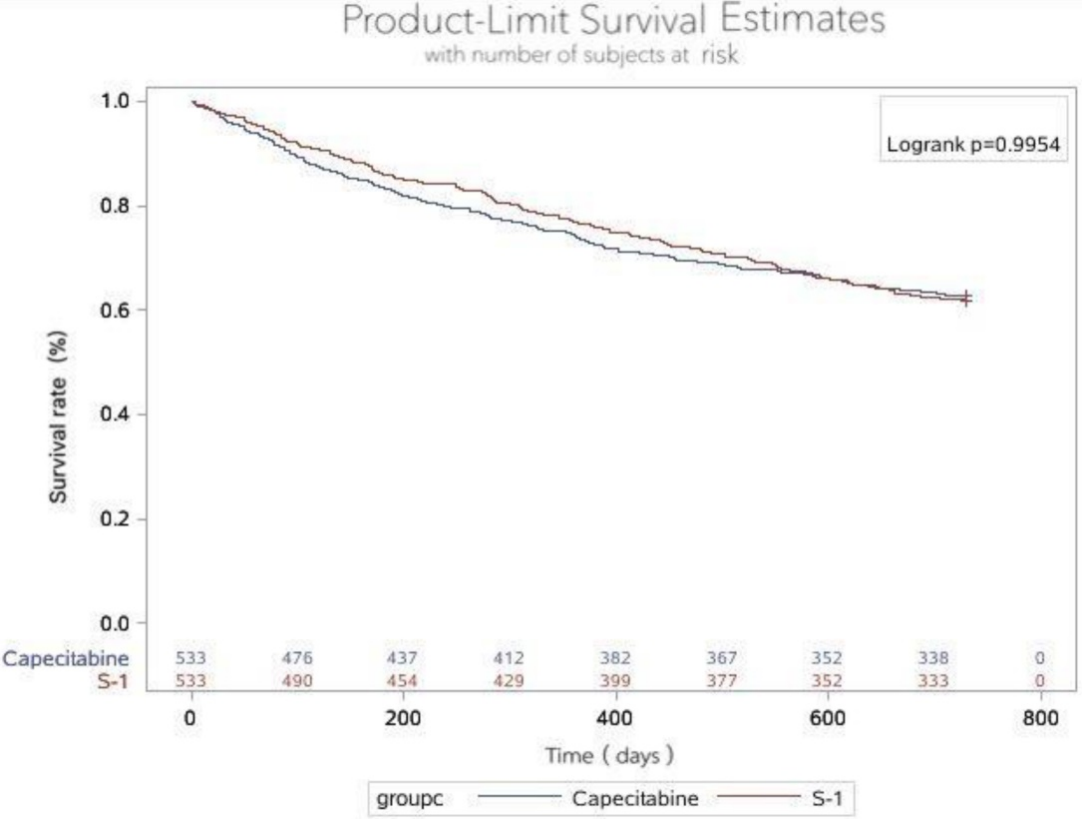
Overall survival in two groups. The 2-year survival rate in the S-1 group and capecitabine group were 61.91 and 62.85%, respectively, of which the difference was no statistically significant (p=0.9954).

We recorded no statistical in 2-years survival rates between two groups (p=0.4295). For the ITT population, 330 patients in the S-1 group still survive in 2-years and 235 patients in the capecitabine (Table 4). The corresponding Kaplan-Meier curves for overall survival (OS) are shown in Figure 3. The HR comparing OS between the two groups was 0.999 (95% Cl 0.822-1.216, p=0.9954) (Table 5).

### Safety

We recorded a similar incidence of adverse events, including leucopenia, neutropenia, anemia, thrombocytopenia (Table 6). The common grade 3 or 4 hematologic toxicity was thrombocytopenia in the S-1 group (8.33% in the S-1 group vs 5.96% in the capecitabine group, P=0.1868). Grade 3 or 4 leucopenia were observed more frequently in the capetabine group (7.63% in the capecitabine group vs 6.45% in the S-1 group,p=0.5990). Hand-foots syndrome (HFS) events were common in all groups (Table 7). As anticipated, HFS of any grade were observed frequently in the capecitabine group (28.71% in the S-1 group vs 46.69% in the capecitabine, p<0.001). Meanwhile, a higher rate of diarrhea was noted in the capecitabine group (16.37% in the S-1 group vs 23.59% in the capecitabine group, p=0.0018). Grade 3 or 4 diarrhea was only observed in the capecitabine group (0 in the S-1 group vs 1.25% in the capecitabine group). Adverse events were mild in two groups and most of them were relieved after appropriate treatment.

## Discussion

The real-world study is a good alternative to get more insight into assessing daily practical question, which avoids the cumbersome trial designs and expensive outgoings. Our real-world study is a retrospective analysis which is comparing S-1 vs capecitabine as the first-line regimen for patients in mCRC. Patients with S-1 or capecitabine monotherapy or doublet chemotherapy including oxaliplatin, irinotecan, raltitrexed. Patients in the S-1 group in the study had 2-years survival rates of 61.91%, which are similar values to those reported in previous trials 18-21. Likewise, the S-1 group in our analysis showed favorable results which are similar to values recorded for capecitabine or 5-Fu treatment in previous studies 2-5,14-17. In addition, several meta-analyses reported that both the S-1 and the capecitabine based regimens were equally active and well tolerated in patients with gastric cancer and CRC 24-26. A meta-analysis compared the efficacy and safety of S-1-based with capecitabine-based regimens in gastrointestinal cancer 25. Results of this meta-analysis indicated that S-1-based and capecitabine-based regimens showed similar efficacy in terms of PFS (HR 0.92, 95% Cl 0.78-1.09, p=0.360), OS (HR 1.01, 95% Cl 0.84-1.21, p=0.949), ORR (HR 1.40,95% Cl 0.87-1.25, p=0.683) and DCR (HR 1.02, 95% Cl 0.94-1,10, p=0.639). There was also no significant difference in toxicity between regimens other than mild more HFS in capecitabine-based regimens. The other analysis focus on patients with locally advances rectal cancer 26. The overall downstaging occurred in 83.3% of the S-1 group and 70.8% of the capecitabine group (p=0.508). The incidence of diarrhea (62.5% vs 33.3%, p=0.014) and hand-foot syndrome (29.2% vs 0%, p=0.016) were higher in capecitabine group. Other adverse events did not differ significantly between the two groups. A phase III trail comparing SOX (S-1 40 mg/m2 twice daily on days 1-14 and oxaliplatin 130 mg/m2 on day 1) vs CapeOX (capecitabine 1000 mg/m2 twice daily on days 1-14 and oxaliplatin 130 mg/m2 on day 1) in Korea reported the efficacy of SOX was also statistically not inferior to that of CapeOX: the median PFS was 7.1 months vs 6.3 months (p=1.0 ), the median OS was 19.0 months 18.4 months (p=0.19) 14. Also, a phase II study reported SOX is an alternative first-line therapy for mCRC 13. SOX and CapeOX both were generally well tolerated, although HFS and Grade 3/4 neutropenia was observed more frequently in the CapeOX group (HFS: 23.8% vs 4.8%, p=0.013; neutropenia: 16.7% vs 2.4%, p=0.026). The efficacy and safety of S-1 were also investment with that of irinotecan to treat colorectal cancer 22, 23. The efficacy of CapeOX was also statistically not inferior to that of CapeIri: the median PFS and OS was 10.4 months (95% Cl 9.0-12.0) and 24.4 months (95% Cl 19.3-30.7) with CapeOX, the median PFS and OS was 12.1 months (95% Cl 10.8-13.2) and 25.5 months (95% Cl 21.0-31.0) with CapeIri. Grade 3/4 diarrhea as predominant toxic effect occurred in 22% of patients with CapeOX and in 16% with CapeIri 22. A study about irinotecan plus S-1 (IRIS) therapy to treat advanced/recurrent colorectal cancer showed the response rate to IRIS was 14.8%, the disease control rate was 60.5%, and the overall survival time was 26.7 months 23. The incidence of grade 3/4 adverse reactions was 17.8%. In comparison to the standard therapy, IRIS had a lower response rate but led to an equivalent overall survival time.

A real-world study about the tolerability of capecitabine monotherapy in mCRC reported that a total of 46.5% of patients experienced HFS and 44.2% experienced a GI event at some time during treatment. Hematological events and cardiotoxicity were rare 6. In a phase II study about SOX regiment with patients in mCRC, a median PFS was 196 days and survival rate (1 year) was 79%. Major grade 3/4 adverse events at the RD were neutropaenia (14%), thrombocytopaenia (28%), and diarrhea (3%) 18. In our study,the rates of adverse events are similar to those of reported clinical trials of S-1 and capecitabine 6,18-21. Thrombocytopenia were observed more frequently in the S-1 group (8.33% in the S-1 group vs 5.96% in the capecitabine group, P=0.1868). Hand-foots syndrome events were common in all groups which was observed frequently in the capecitabine group (28.71% in the S-1 group vs 46.69% in the capecitabine, p<0.001). Meanwhile, a higher rate of diarrhea was noted in the capecitabine group (16.37% in the S-1 group vs 23.59% in the capecitabine group, p=0.0018). Grade 3 or 4 diarrhea was only observed in the capecitabine group (0 in the S-1 group vs 1.25% in the capecitabine group). However, there were significant limitations of this study, including its retrospective nature and the quality of the real-world data we were able to obtain.

## Conclusion

Compared to capecitabine, S-1 had a similar 2-year OS rate but had a lower incidence of adverse events in the real clinical setting. So, S-1 could be a good choice in the first-treatment of patients with metastatic colorectal cancer in real-world in China.

## Figures and Tables

**Table 1:** Patient Demographics.

Characteristics	S-1 (%) [N=546]	Capecitabine (%) [N=821]	P-value
**Gender**			0.1144
Male	315(57.7)	509(62.0)	
Female	231(42.3)	312(38.0)	
**Age[Year]**			0.8375
Mean ± SD	58.7±11.53	58.8±11.47	
**Age[Year]**			0.7479
≦60	283(51.8)	417(50.8)	
>60	263(48.2)	404(49.2)	
**BMI Index**			0.1593
<18.5	32(5.98)	66(8.21)	
18.5~24	259(48.41)	375(46.64)	
24~28	175(32.71)	282(35.07)	
>28	69(12.9)	81(10.08)	
Missing=28	11	17	
**KPS Score**			0.7281
60	6(1.17)	15(1.98)	
70	27(5.26)	36(4.76)	
80	299(58.29)	442(58.47)	
90	181(35.28)	264(34.79)	
Missing=97	33	64	

**Table 2:** The Pre- and Post- matching patients in two groups.

Item	Capecitabine	S-1
Total Subjects	800	533
Matched Subjects	533	533
Unmatched Subjects	267	0
Excluded Subjects	0	0

**Table 3:** Patient Demographics.

Characteristics	S-1 (%) [N=533]	Capecitabine (%) [N=533]	P-value
**Gender**			0.1338
Male	306(57.41)	331(62.10)	
Female	227(42.59)	202(37.90)	
**Age[Year]**			0.4005
Mean ± SD	58.7±11.60	59.4±11.55	
**Age[Year]**			0.5004
≤60	276(51.78)	264(49.53)	
>60	257(48.22)	269(50.47)	
**BMI Index**			0.1974
<18.5	32(6.13)	40(7.65)	
18.5~24	254(48.66)	251(47.99)	
24~28	169(32.38)	184(35.18)	
>28	67(12.83)	48(9.18)	
Missing=21	11	10	
**KPS Score**			0.6839
60	6(1.20)	10(2.03)	
70	27(5.40)	23(4.67)	
80	295(59.00)	297(60.24)	
90	172(34.40)	163(33.06)	
Missing=73	33	40	

**Table 4:** The 2-year survival rates in two groups.

Characteristics	S-1 (%) [N=533]	Capecitabine (%) [N=533]	P-value
**Survival status**			0.4295*
Survival	330(61.91)	335(62.85)	
Death	203(38.09)	198(37.15)	

**Table 5:** Results of Hazard Ratio using group as response variable.

Risk factor	Hazard Ratio	95%CI	P value
**Group**			0.9954
S-1 vs Capecitabine	0.999	(0.822,1.216)	

**Table 6:** Blood Routine Indicators.

Characteristics	S-1 (%) [N=533]	Capecitabine (%) [N=533]	P-value
**White Blood Count**			0.5990
0	244(46.30)	229(43.71)	
I~II	249(47.25)	255(48.66)	
III~IV	34(6.45)	40(7.63)	
Missing=15	6	9	
**Platelets Count**			0.1868
0	278(53.88%)	304(58.46)	
I~II	195(37.79)	185(35.58)	
III~IV	43(8.33)	31(5.96)	
Missing=30	17	13	
**Neutrophil Count**			0.0516
0	280(53.64)	256(49.33)	
I~II	206(39.46)	239(46.05)	
III~IV	36(6.90)	24(4.62)	
Missing=25	11	14	

**Table 7:** Other Indicators.

Characteristics	S-1 (%) [N=533]	Capecitabine (%) [N=533]	P-value
**Electrocardiogram**			0.2489
Normal	407(80.28)	388(77.14)	
Abnormal	100(19.72)	115(22.86)	
Missing=56	26	30	
**Liver function**			0.1698
0	324(62.91)	292(57.37)	
I~II	186(36.12)	210(41.26)	
III~IV	5(0.97)	7(1.38)	
Missing=42	18	24	
**Renal Function**			0.0962
0	453(88.65)	436(84.99)	
I~II	58(11.35)	77(15.01)	
Missing	22	20	
**Nausea and Vomiting**			0.0991
0	318(65.43)	284(58.80)	
I~II	154(31.69)	182(37.68)	
III~IV	14(2.88)	17(3.52)	
Missing=97	47	50	
**Diarrhea**			0.0018
0	378(83.63)	366(76.41)	
I~II	74(16.37)	107(22.34)	
III~IV	0	6(1.25)	
Missing=105	51	54	
**Hand-foot Syndrome**			<0.001
0	355(71.29)	258(53.31)	
I~II	136(27.31)	192(39.67)	
III~IV	7(1.40)	34(7.02)	
Missing=84	35	49	
